# Knowledge, attitudes, practices on hepatitis C and HCV screening: an Italian cross-sectional study

**DOI:** 10.1093/eurpub/ckac131.130

**Published:** 2022-10-25

**Authors:** G Lo Moro, G Scaioli, R Frattin, L Guastavigna, L Vola, F Bert, R Siliquini

**Affiliations:** 1 Department of Public Health Sciences, University of Turin, Turin, Italy; 2 A.O.U. City of Health and Science of Turin, Turin, Italy

## Abstract

**Background:**

Hepatitis C has the highest burden in the Eastern Mediterranean Region and European Region. This study aimed to explore knowledge, attitudes and practices towards hepatitis C and HCV screening, which has been large-scale implemented in Italy recently.

**Methods:**

An online nationwide cross-sectional survey was conducted (Italy, December 2021). Five outcomes were used: HCV knowledge (from 0 to 100%: 100% represents the lowest knowledge); not being aware of the screening; wrong attitudes in case of contact with positive people (from 0 to 5: 5 represents more wrong attitudes); having performed an HCV test; sharing of blood-contaminated objects. Multivariable regressions were run.

**Results:**

Participants were 813 (74.7% females; mean age 37 years, SD 12.4). The median score of HCV knowledge was 20% (IQR 16-24). There was a positive correlation between poor knowledge and wrong attitudes (p = 0.001). People who underwent an HCV test were less likely to have poor knowledge (p = 0.040). The 23.2% was not aware of screening existence. People who had a postgraduate degree were less likely of not being aware (p = 0.004). Investigating attitude score, the median was 0 (IQR 0-1). Increasing age was associated with wrong attitudes (p = 0.020). The 43.4% underwent an HCV test. This likelihood was greater for: residence in a municipality with more than 50000 inhabitants (p = 0.032); having at least one child (p = 0.009); considering oneself at risk (p = 0.004); being informed about HCV (p < 0.001). The 31.8% shared objects. Increasing age was associated with reduced odds of sharing (p = 0.033). The 72.4% would like to receive more information on HCV, preferring brochures and short videos.

**Conclusions:**

This study showed good knowledge and attitudes, with a substantial frequency of individuals who never underwent an HCV test or shared contaminated objects. It also suggested brochures and short videos may be the most acceptable ways to implement awareness campaigns in comparable European contexts.

**Key messages:**

• Italian general population had good knowledge and attitudes towards hepatitis C and its screening, while it showed poor practices.

• Most of participants were willing to receive more information on HCV, especially through brochures and short videos.

